# Supplementary material for: Effects of Caffeine Dose and Administration Method on Time-Trial Performance: A Systematic Review and Network Meta-Analysis
Source: Nutrients. 2025 Dec 3;17(23):3792. doi: 10.3390/nu17233792 (PMC12694421; doi:10.3390/nu17233792)
Supplement: Supplementary file 1 [file nutrients-17-03792-s001.zip › nutrients-3999783-supplementary.pdf]

Supplemental files

Contents

Supplement S1: PRISMA NMA Checklist 2

Supplement S2: Search strategy 6

Supplement S3: Study characteristics 7

Supplement S4: Risk of bias assessments 15

Supplement S5: Pairwise meta-analyse 17

Supplement S6: Heterogeneity assessments 18

Supplement S7: Intransitivity assessments 20

Supplement S8: Inconsistency (incoherence) assessments 21

Supplement S9: Forest plot of all studies 24

Supplement S10: Comparison-adjusted funnel plots 25

Supplement S11: Trim and fill analysis 26

Supplement S12: Network meta-regression 27

Supplement S1: PRISMA NMA Checklist

Section/Topic	Item #	Checklist Item	Reported on Page #
**TITLE**			
Title	1	Identify the report as a systematic review *incorporating a network meta-analysis (or related form of meta-analysis).*	1
**ABSTRACT**			
Structured summary	2	Provide a structured summary including, as applicable:<br>**Background:** main objectives<br>**Methods:** data sources; study eligibility criteria, participants, and interventions; study appraisal; and *synthesis methods, such as network meta-analysis.*<br>**Results:** number of studies and participants identified; summary estimates with corresponding confidence/credible intervals; *treatment rankings may also be discussed. Authors may choose to summarize pairwise comparisons against a chosen treatment included in their analyses for brevity.*<br>**Discussion/Conclusions:** limitations; conclusions and implications of findings.<br>**Other:** primary source of funding; systematic review registration number with registry name.	1
**INTRODUCTION**			
Rationale	3	Describe the rationale for the review in the context of what is already known, *including mention of why a network meta-analysis has been conducted.*_	1-2
Objectives	4	Provide an explicit statement of questions being addressed, with reference to participants, interventions, comparisons, outcomes, and study design (PICOS).	1-2
**METHODS**			
Protocol and registration	5	Indicate whether a review protocol exists and if and where it can be accessed (e.g., Web address); and, if available, provide registration information, including registration number.	2-3
Eligibility criteria	6	Specify study characteristics (e.g., PICOS, length of follow-up) and report characteristics (e.g., years considered, language, publication status) used as criteria for eligibility, giving rationale. *Clearly describe eligible treatments included in the treatment network, and note whether any have been clustered or merged into the same node (with justification).*_	3
Information sources	7	Describe all information sources (e.g., databases with dates of coverage, contact with study authors to identify additional studies) in the search and date last searched.	3
Search	8	Present full electronic search strategy for at least one database, including any limits used, such that it could be repeated.	3， Supplement

			2
Study selection	9	State the process for selecting studies (i.e., screening, eligibility, included in systematic review, and, if applicable, included in the meta-analysis).	3
Data collection process	10	Describe method of data extraction from reports (e.g., piloted forms, independently, in duplicate) and any processes for obtaining and confirming data from investigators.	3
Data items	11	List and define all variables for which data were sought (e.g., PICOS, funding sources) and any assumptions and simplifications made.	3
**Geometry of the network**	**S1**	Describe methods used to explore the geometry of the treatment network under study and potential biases related to it. This should include how the evidence base has been graphically summarized for presentation, and what characteristics were compiled and used to describe the evidence base to readers.	4
Risk of bias within individual studies	12	Describe methods used for assessing risk of bias of individual studies (including specification of whether this was done at the study or outcome level), and how this information is to be used in any data synthesis.	4
Summary measures	13	State the principal summary measures (e.g., risk ratio, difference in means). *Also describe the use of additional summary measures assessed, such as treatment rankings and surface under the cumulative ranking curve (SUCRA) values, as well as modified approaches used to present summary findings from meta-analyses.*	4
Planned methods of analysis	14	Describe the methods of handling data and combining results of studies for each network meta-analysis. This should include, but not be limited to:<br>• *Handling of multi-arm trials;*<br>• *Selection of variance structure;*<br>• *Selection of prior distributions in Bayesian analyses; and*<br>• *Assessment of model fit.*	4
**Assessment of Inconsistency**	**S2**	Describe the statistical methods used to evaluate the agreement of direct and indirect evidence in the treatment network(s) studied. Describe efforts taken to address its presence when found.	4
Risk of bias across studies	15	Specify any assessment of risk of bias that may affect the cumulative evidence (e.g., publication bias, selective reporting within studies).	4
Additional analyses	16	Describe methods of additional analyses if done, indicating which were pre-specified. This may include, but not be limited to, the following:<br>• Sensitivity or subgroup analyses;<br>• Meta-regression analyses;<br>• *Alternative formulations of the treatment network; and*<br>• *Use of alternative prior distributions for Bayesian analyses (if applicable).*_	4
**RESULTS†**			

Study selection	17	Give numbers of studies screened, assessed for eligibility, and included in the review, with reasons for exclusions at each stage, ideally with a flow diagram.	4-5，Figure 1
**Presentation of network structure**	**S3**	Provide a network graph of the included studies to enable visualization of the geometry of the treatment network.	Figure 2
**Summary of network geometry**	**S4**	Provide a brief overview of characteristics of the treatment network. This may include commentary on the abundance of trials and randomized patients for the different interventions and pairwise comparisons in the network, gaps of evidence in the treatment network, and potential biases reflected by the network structure.	5-6
Study characteristics	18	For each study, present characteristics for which data were extracted (e.g., study size, PICOS, follow-up period) and provide the citations.	5-6, Supplement 3
Risk of bias within studies	19	Present data on risk of bias of each study and, if available, any outcome level assessment.	5-6, Supplement 3
Results of individual studies	20	For all outcomes considered (benefits or harms), present, for each study: 1) simple summary data for each intervention group, and 2) effect estimates and confidence intervals. *Modified approaches may be needed to deal with information from larger networks.*	*Not applicable*
Synthesis of results	21	Present results of each meta-analysis done, including confidence/credible intervals. *In larger networks, authors may focus on comparisons versus a particular comparator (e.g., placebo or standard care), with full findings presented in an appendix. League tables and forest plots may be considered to summarize pairwise comparisons.* If additional summary measures were explored (such as treatment rankings), these should also be presented.	5-6, Table 1, Figure 2, Supplement 5, Supplement 9
**Exploration for inconsistency**	**S5**	Describe results from investigations of inconsistency. This may include such information as measures of model fit to compare consistency and inconsistency models, *P* values from statistical tests, or summary of inconsistency estimates from different parts of the treatment network.	5-6, Supplement 6-8
Risk of bias across studies	22	Present results of any assessment of risk of bias across studies for the evidence base being studied.	5-6, Supplement 4
Results of additional analyses	23	Give results of additional analyses, if done (e.g., sensitivity or subgroup analyses, meta-regression analyses, *alternative network geometries studied, alternative choice of prior distributions for Bayesian analyses,* and so forth).	5-6, Supplement 10-12
**DISCUSSION**			
Summary of evidence	24	Summarize the main findings, including the strength of evidence for each main outcome; consider their relevance to key groups (e.g., healthcare providers, users, and policy-makers).	8

Limitations	25	Discuss limitations at study and outcome level (e.g., risk of bias), and at review level (e.g., incomplete retrieval of identified research, reporting bias). *Comment on the validity of the assumptions, such as transitivity and consistency. Comment on any concerns regarding network geometry (e.g., avoidance of certain comparisons).*	8-10
Conclusions	26	Provide a general interpretation of the results in the context of other evidence, and implications for future research.	10
**FUNDING**			
Funding	27	Describe sources of funding for the systematic review and other support (e.g., supply of data); role of funders for the systematic review. This should also include information regarding whether funding has been received from manufacturers of treatments in the network and/or whether some of the authors are content experts with professional conflicts of interest that could affect use of treatments in the network.	10

PICOS = population, intervention, comparators, outcomes, study design.

* Text in italics indicate wording specific to reporting of network meta-analyses that has been added to guidance from the PRISMA statement.

† Authors may wish to plan for use of appendices to present all relevant information in full detail for items in this section.

Supplement S2: Search strategy

Search level	Search terms with Boolean operators
Search#1	"caffeine*" OR "coffein*" OR "coffee" OR "chewing gum" OR "gum chewing" OR "caffeine capsule" OR "caffeine mouth rinse"
Search#2	#1 AND ("Endurance" OR "exercise" OR "running" OR "Performance" OR "Athletic performance" OR "aerobic capacity" OR "Time trial" OR "time to exhaustion")
Search#3	#2 NOT ("patient" OR "patients")

Supplement S3: Study characteristics

Study	Sample	Age (years)	$\dot{V}O_{2max}$ (ml/min/kg)	Caffeine does (mg/kg)	Timing (min)	Habitual caffeine intake (mg/day)	Caffeine withdrawal (h)	Caffeine form	Mode of exercise	Performance levels	Exercise protocol	Total exercise duration (min)	performance % change (Time)	performance % change (MPO)
Ghaderi et al. a 2013	15 M	21 ± 4	n/a	5	5	n/a	24	Gum	Running	Recreational	1.5 Km TT	5.7	-2.8%	n/a
Ghaderi et al. b 2013	15 M	21 ± 4	n/a	5	35	n/a	24	Capsules	Running	Recreational	1.5 Km TT	5.7	-2.3%	n/a
Skinner et al. a 2010	10 M	20 ± 1	58.1 ± 6.8	2	60	n/a	24	Capsules	Rowing	Competitive	2 Km TT	6.7	-0.3%	1.1%
Skinner et al. b 2010	10 M	20 ± 1	58.1 ± 6.8	4	60	n/a	24	Capsules	Rowing	Competitive	2 Km TT	6.7	-0.7%	1.7%
Skinner et al. c 2010	10 M	20 ± 1	58.1 ± 6.8	6	60	n/a	24	Capsules	Rowing	Competitive	2 Km TT	6.7	-0.3%	0.9%
Carr et al. 2011	6 M / 2 F	n/a	n/a	6	30	n/a	48	Capsules	Rowing	Recreational	2 Km TT	6.7	-0.7%	2.3%
Gharaat et al. a 2020	9 M	18 ± 1	51.4 ± 3.9	3	30	< 50	n/a	Capsules	Rowing	Recreational	2 Km TT	6.9	-1.0%	3.4%
Gharaat et al. b 2020	9 M	18 ± 1	51.4 ± 3.9	6	30	< 50	n/a	Capsules	Rowing	Recreational	2 Km TT	6.8	-2.7%	7.6%
Khcharem et al. 2021	13 M	21 ± 1	51.3 ± 6.1	3	60	28.2 ± 11.6	48	Capsules	Running	Recreational	3 Km TT	10.1	-1.1%	n/a
Santos et al. 2013	8 M	32 ± 5	57.5 ± 5.8	5	60	n/a	24	Capsules	Cycling	Recreational	4 Km TT	6.8	-2.3%	5.9%
Felippe et al. 2018	11 M	34 ± 4	55 ± 4	5	60	n/a	24	Capsules	Cycling	Recreational	4 Km TT	6.6	-1.6%	12.0%

Tomazini et al. 2022	11 M	33 ± 7	56.1 ± 13.2	5	60	171 ± 143	24	Capsules	Cycling	Recreational	4 Km TT	6.1	-0.9%	2.1%
Viana et al. 2020	9 M	32 ± 8	55 ± 6.1	6	60	50 - 200	24	Capsules	Cycling	Recreational	4 Km TT	5.8	-2.0%	7.6%
Couto et al. 2022	9 M	32 ± 6	55.2 ± 5.7	5	60	86 ± 71	24	Capsules	Cycling	Recreational	4 Km TT	6.1	-1.8%	n/a
Khcharem et al. 2020	10 M	21 ± 1	51.3 ± 6.4	5	60	28.2 ± 11.6	n/a	Capsules	Running	Recreational	8 Km TT	33.2	-2.4%	n/a
Griest et al. a 2023	11 M / 4 F	39 ± 15	54.2 ± 6.2	6	60	< 500	0	Capsules	Cycling	Recreational	10 Km TT	18.2	-0.4%	1.9%
Griest et al. b 2023	11 M / 4 F	39 ± 15	54.2 ± 6.2	6	60	< 500	24	Capsules	Cycling	Recreational	10 Km TT	18.1	-4.3%	8.5%
Graham-Paulson et al. 2016	11 M	24 ± 4	42.9 ± 7.3	4	45	160 ± 160	24	Capsules	Cycling	Recreational	30 min 65% $\dot{V}O_{2max}$; 10 Km TT	46.6	-2.1%	n/a
Saunders et al. 2016	42 M	37 ± 8	50 ± 6.8	6	60	n/a	n/a	Capsules	Cycling	Recreational	16 Km TT	n/a	n/a	2.6%
Bortolotti et al. 2014	13 M	26 ± 10	n/a	6	60	n/a	48	Capsules	Cycling	Recreational	20 Km TT	36.4	-0.5%	-1.1%
Glaister et al. 2015	14 F	31 ± 7	52.3 ± 4.9	5	60	249 ± 131	24	Capsules	Cycling	Recreational	20 Km TT	34.6	-2.1%	5.4%
Skinner et al. 2013	14 M	31 ± 5.2	69.5 ± 6.1	6	60	n/a	48	Capsules	Cycling	Competitive	40 Km TT	58.5	-1.1%	n/a

Potgieter et al. a 2018	14 M	37 ± 10	n/a	6	60	413 ± 5.5	48	Capsules	Triathlon	Recreational	1.5 Km swim; 40 Km cycle; 10 Km run	144.5	-1.8%	n/a
Potgieter et al. b 2018	12 F	37 ± 10	n/a	6	60	413 ± 5.5	48	Capsules	Triathlon	Recreational	1.5 Km swim; 40 Km cycle; 10 Km run	157.8	-0.9%	n/a
Desbrow et al. a 2012	16 M	32 ± 8	60.4 ± 4.1	3	90	210 ± 115	24	Capsules	Cycling	Competitive	0.75 × W_{max} × 3600 KJ TT	62.3	-4.2%	n/a
Desbrow et al. b 2012	16 M	32 ± 8	60.4 ± 4.1	6	90	210 ± 115	24	Capsules	Cycling	Competitive	0.75 × W_{max} × 3600 KJ TT	63.2	-2.8%	n/a
Irwin et al. a 2011	12 M	28 ± 5	63.7 ± 7.4	3	90	240 ± 162	96	Capsules	Cycling	Recreational	0.75 × $\dot{V}O_{2max}$ × 1100 KJ TT	58.1	-3.0%	3.3%
Irwin et al. b 2011	12 M	28 ± 5	63.7 ± 7.4	3	90	240 ± 162	n/a	Capsules	Cycling	Recreational	0.75 × $\dot{V}O_{2max}$ × 1100 KJ TT	57.7	-3.5%	3.9%
Wallman et al. 2010	10 F	22 ± 2	n/a	6	60	< 100	48	Capsules	Cycling	Recreational	15 min 65% HR_{max}; 10 min TT	25.0	n/a	5.6%
Barreto et al. 2020	15 M	38 ± 8	51.1 ± 7.1	6	60	< 200	24	Capsules	Cycling	Recreational	25 min TT	25.0	n/a	2.4%
Conway et al. 2002	8 M	25 ± 5	72 ± 3.9	6	60	< 250	48	Capsules	Cycling	Competitive	90 min 70% $\dot{V}O_{2max}$; 30 min TT	113.8	-15.9%	n/a

Laurence et al. 2012	12 M	25 ± 2	n/a	6	60	n/a	48	Capsules	Cycling	Recreational	30 min TT	30.0	n/a	0.3%
Ruiz-Moreno et al. 2022	11 M / 4 F	29 ± 6	49.3 ± 10.4	3	60	n/a	n/a	Capsules	Cycling	Recreational	60 min TT	60.0	n/a	10.3%
Vinícius et al. 2025 a	13 M	36.2 ± 3.3	n/a	5	50	458.6 ± 375.5	n/a	Capsules	Cycling	Competitive	4 Km TT		-1.9%	n/a
Vinícius et al. 2025 b	13 M	36.2 ± 3.3	n/a	5	50	458.6 ± 375.5	n/a	Capsules	Cycling	Competitive	4 Km TT	6.2	-0.5%	n/a
Vinícius et al. 2025 c	13 M	36.2 ± 3.3	n/a	5	80	458.6 ± 375.5	n/a	Capsules	Cycling	Competitive	4 Km TT	6.2	-0.5%	n/a
Vinícius et al. 2025 d	13 M	36.2 ± 3.3	n/a	5	80	458.6 ± 375.5	n/a	Capsules	Cycling	Competitive	4 Km TT	6.2	-0.5%	n/a
Farhadi et al. a 2011	15 M	21 ± 4	n/a	3	5	n/a	24	Gum	Running	Recreational	1.5 Km TT	5.6	-4.3%	n/a
Farhadi et al. b 2011	15 M	21 ± 4	n/a	4	5	n/a	24	Gum	Running	Recreational	1.5 Km TT	5.6	-4.0%	n/a
Farhadi et al. c 2011	15 M	21 ± 4	n/a	5	5	n/a	24	Gum	Running	Recreational	1.5 Km TT	5.7	-2.8%	n/a
Whalley. a 2020	10 M / 4 F	40 ± 8	n/a	4	10	< 400	24	Gum	Running	Recreational	5 Km TT	20.5	-1.1%	n/a
Whalley. b 2020	10 M / 4	40 ± 8	n/a	4	60	< 400	24	Capsules	Running	Recreational	5 Km TT	20.3	-2.1%	n/a

	F													
Lynn et al. 2024	36 M	33.7 ± 10.7	n/a	4	30	n/a	24	Gum	Running	Recreational	5 Km TT	22.0	-3.6%	n/a
Oberlin-Brown et al. 2016	11 M	32 ± 7	60.2 ± 4	3	5	n/a	24	Gum	Cycling	Recreational	20 Km TT	32.3	-0.4%	n/a
Lane et al. a 2014	12 M	31 ± 7	71.6 ± 4.6	3	40	n/a	24	Gum	Cycling	Competitive	43.83 Km TT	62.7	-1.3%	3.2%
Lane et al. b 2014	12 F	28 ± 6	59.9 ± 5.1	3	40	n/a	24	Gum	Cycling	Competitive	29.53 KM TT	50.8	-1.6%	4.2%
Ryan et al. a 2013	8 M	25 ± 5	50 ± 5	4	5	n/a	n/a	Gum	Cycling	Recreational	15 min 75% $\dot{V}O_{2max}$; 700 KJ TT	53.7	-4.9%	n/a
Ryan et al. b 2013	8 M	25 ± 5	50 ± 5	4	60	n/a	n/a	Gum	Cycling	Recreational	15 min 75% $\dot{V}O_{2max}$; 700 KJ TT	56.8	2.7%	n/a
Ryan et al. c 2013	8 M	25 ± 5	50 ± 5	4	120	n/a	n/a	Gum	Cycling	Recreational	15 min 75% $\dot{V}O_{2max}$; 700 KJ TT	57.6	4.7%	n/a
Algrain et al. a 2016	11	23 ± 2	31.9 ± 1.8	4	5	< 300	48	Gum	Cycling	Recreational	15 min 75% $\dot{V}O_{2max}$; 15 min TT	30.0	n/a	2.3%
Algrain et al.b 2016	9	26 ± 1	31.7 ± 2.7	4	5	< 300	48	Gum	Cycling	Recreational	15 min 75% $\dot{V}O_{2max}$; 15 min TT	30.0	n/a	3.6%
O’Rourke et al. a 2008	15 M	32 ± 8	n/a	5	60	n/a	48	Capsules	Running	Recreational	5 Km TT	17.5	-1.0%	n/a

O'Rourke et al. b 2008	15 M	29 ± 5	n/a	5	60	n/a	48	Capsules	Running	Recreational	5 Km TT	21.4	-0.9%	n/a
Christensen et al. 2014	11 M / 1 F	25 ± 3	n/a	3	45	< 200	36	Capsules	Rowing	Competitive	6 min TT	6.0	n/a	1.8%
Jenkins et al. a 2008	13 M	26 ± 6	55.2 ± 7.2	2	60	132 ± 41	48	Capsules	Cycling	Competitive	15 min 80% $\dot{V}O_{2max}$; 15 min TT	30.0	n/a	3.9%
Jenkins et al. b 2008	13 M	26 ± 6	55.2 ± 7.2	3	60	132 ± 41	48	Capsules	Cycling	Competitive	15 min 80% $\dot{V}O_{2max}$; 15 min TT	30.0	n/a	3.0%
Boat et al. 2021	15 M	22 ± 2	n/a	3	10 s	n/a	24	Mouth-rinse	Cycling	Recreational	10 Km TT	16.5	-0.6%	n/a
Figueiredo et al. 2021	8 M / 2 F	30 ± 6	n/a	4	10 s	n/a	48	Mouth-rinse	Running	Recreational	10 Km TT	47.5	0.8%	n/a
Lesniak et al. 2016	7 F	21 ± 1	37.99 ± 0.92	5	5 s	n/a	24	Mouth-rinse	Cycling	Recreational	0.6 × W_{max} × 3600 KJ TT	61.6	0.1%	-0.4%
Bottoms et al. 2014	12 M	20 ± 1	n/a	2	5 s	n/a	24	Mouth-rinse	Cycling	Recreational	30 min TT	30.0	n/a	6.4%
Doering et al. 2014	10 M	32 ± 7	59.8 ± 3.5	4	10 s	n/a	24	Mouth-rinse	Cycling	Recreational	60 min TT	65.3	-0.6%	n/a

Acker-Hewitt et al. 2012	12 M	28 ± 9	66 ± 9	6	60	n/a	24	Capsules	Cycling	Competitive	20 min 60% W_{max}; 20 Km TT	63.6	-1.4%	3.80%
Spence et al. 2013	10 M	30 ± 2	59 ± 2	2.5	60	n/a	48	Capsules	Cycling	Competitive	10 min 55% W_{max}; 10 Km TT	28.0	-4.5%	9.30%
Goncalves et al. a 2017	14 M	34 ± 9	50 ± 9	6	60	58 ± 29	24	Capsules	Cycling	Competitive	0.85 × W_{max} ×1500 KJ TT	30.3	−1.6%	n/a
Goncalves et al. b 2017	12 M	37 ± 7	51 ± 6	6	60	143 ± 25	24	Capsules	Cycling	Competitive	0.85 × W_{max} ×1500 KJ TT	29.7	−3.7%	n/a
Goncalves et al. c 2017	14 M	37 ± 8	51 ± 8	6	60	351 ± 139	24	Capsules	Cycling	Competitive	0.85 × W_{max} ×1500 KJ TT	30.4	−1.4%	n/a
Kilding et al. 2013	10 M	24 ± 5	n/a	3	60	n/a	24	Capsules	Cycling	Competitive	20 min 60–65% MPO; 3 Km TT	23.8	-1.0%	2.10%
Quinlivan et al. 2015	11 M	32 ± 6	61 ± 8	3	90	271 ± 295	24	Capsules	Cycling	Competitive	0.75 × W_{max} ×3600 KJ TT	62.6	-3.1%	2.80%
Larson et al. 2018	9 M	34 ± 11	61 ± 6	5	60	100–400	12	Capsules	Cycling	Competitive	20 Km TT	33.2	-3.9%	4.40%
Santos et al. a 2020	8 M	35 ± 5	57 ± 8	5	60	n/a	24	Capsules	Cycling	Competitive	4 Km TT	6.1	-1.5%	4.30%
Santos et al. b 2020	8 M	35 ± 4	49 ± 10	5	60	n/a	24	Capsules	Cycling	Competitive	4 Km TT	6.7	-2.5%	6.50%

“a”, “b”, “c”, “d” represents the number of trials of the same study. F: Females, M: Males, KJ: Kilojoules. MPO: mean power output, n/a: no available, Time: completion time, Timing: time of ingestion before the experimental session, TT: time trail, $\dot{V}O_{2max}$: maximal oxygen uptake, W_{max}: work rate max.

Supplement S4: Risk of bias assessments

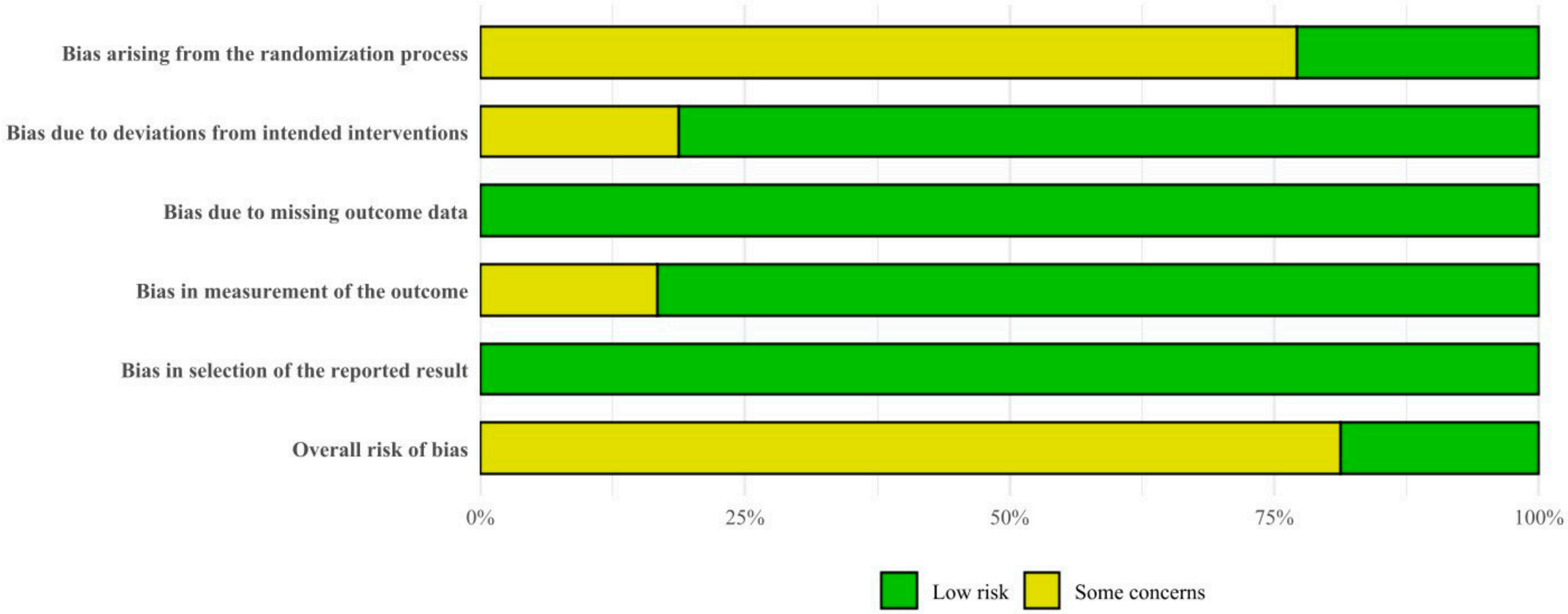

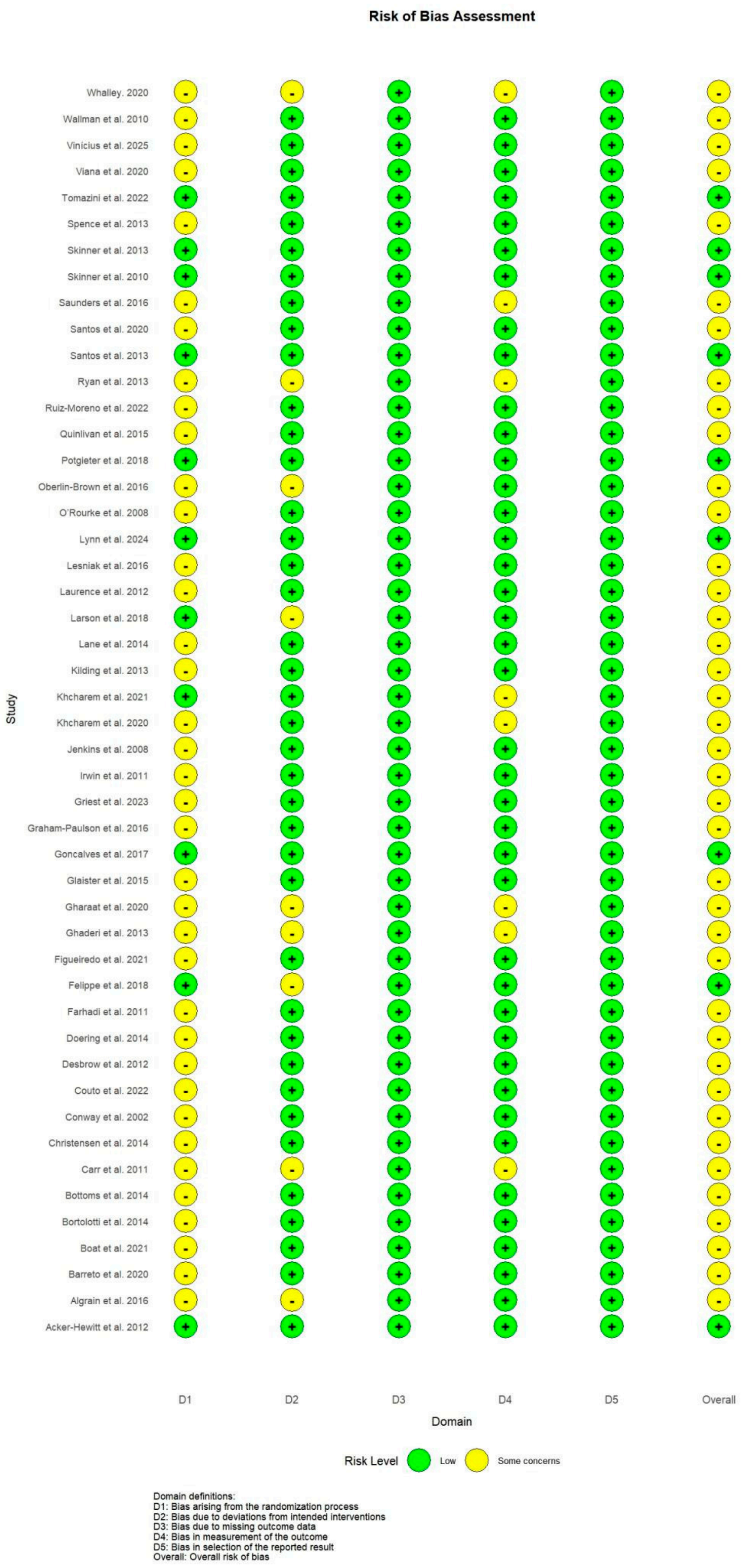
Risk of Bias Assessment
Study
Whalley. 2020
Wallman et al. 2010
Vinicius et al. 2025
Viana et al. 2020
Tomazini et al. 2022
Spence et al. 2013
Skinner et al. 2013
Skinner et al. 2010
Saunders et al. 2016
Santos et al. 2020
Santos et al. 2013
Ryan et al. 2013
Ruiz-Moreno et al. 2022
Quinlivan et al. 2015
Potgieter et al. 2018
Oberlin-Brown et al. 2016
O'Rourke et al. 2008
Lynn et al. 2024
Lesniak et al. 2016
Laurence et al. 2012
Larson et al. 2018
Lane et al. 2014
Kilding et al. 2013
Khcharem et al. 2021
Khcharem et al. 2020
Jenkins et al. 2008
Irwin et al. 2011
Griest et al. 2023
Graham-Paulson et al. 2016
Goncalves et al. 2017
Glaister et al. 2015
Gharaat et al. 2020
Ghaderi et al. 2013
Figueiredo et al. 2021
Felippe et al. 2018
Farhadi et al. 2011
Doering et al. 2014
Desbrow et al. 2012
Couto et al. 2022
Conway et al. 2002
Christensen et al. 2014
Carr et al. 2011
Bottoms et al. 2014
Bortolotti et al. 2014
Boat et al. 2021
Barreto et al. 2020
Algrain et al. 2016
Acker-Hewitt et al. 2012
D1
D2
D3
D4
D5
Overall
Domain
Risk Level
Low
Some concerns
Domain definitions:
D1: Bias arising from the randomization process
D2: Bias due to deviations from intended interventions
D3: Bias due to missing outcome data
D4: Bias in measurement of the outcome
D5: Bias in selection of the reported result
Overall: Overall risk of bias

Supplement S5: Pairwise meta-analyse

Comparison	Number of studies	SMD (95% CrI)	I^2
**Completion time**			
Low-dose gum vs Placebo	4	-0.34 (-0.74, 0.05)	0
Low-dose capsules vs Placebo	9	**-0.34 (-0.62, -0.06)**	0
Moderate-dose capsules vs Placebo	35	**-0.31 (-0.44, -0.17)**	0
Moderate-dose gum vs Placebo	8	**-0.28 (-0.57, -0.01)**	0
Low-dose mouth rinse vs Placebo	1	-0.26 (-0.98, 0.46)	0
Moderate-dose mouth rinse vs Placebo	3	-0.00 (-0.54, 0.53)	0
**Mean power output**			
Low-dose mouth rinse vs Placebo	1	0.39 (-0.42, 1.19)	0
Low-dose capsules vs Placebo	8	**0.38 (0.09, 0.67)**	0
Moderate-dose capsules vs Placebo	20	**0.29 (0.10, 0.49)**	0
Low-dose gum vs Placebo	5	0.17 (-0.41, 0.75)	0
Moderate-dose gum vs Placebo	3	0.24 (-0.25, 0.73)	0
Moderate-dose mouth rinse vs Placebo	1	-0.05 (-1.10, 1.00)	0

Note: Bolded numbers indicate the presence of significance. SMD: standardized mean differences. CrI: credible interval.

Supplement S6: Heterogeneity assessments

Outcomes	Design-based Q statistic, comparisons, and overall statement	Q statistic	Degree of freedom	*P* -value
**Completion time**	**Design-specific decomposition of within-designs Q statistic**			
	Placebo: Moderate-dose gum	4.41	5	0.4921
	Placebo: Low-dose gum	1.20	3	0.7526
	Placebo: Moderate-dose capsules	0.17	2	0.9187
	Placebo: Moderate-dose mouth rinse	0.07	2	0.9670
	Placebo: Low-dose capsules	1.10	8	0.9976
	Placebo: Moderate-dose capsules	11.53	32	0.9997
	**Between-designs Q statistic after detaching of single designs**			
	Placebo: Moderate-dose capsules	0.17	1	0.6759
	Placebo: Moderate-dose gum	0.20	1	0.6536
	Placebo: Moderate-dose capsules: Moderate-dose gum	0.00	0	--
	Q statistic to assess consistency under the assumption of a full design-by-treatment interaction random effects model:			

	between designs Q statistic, 0.27; degree of freedom, 2; *p*-value, 0.8744; tau. within, 0; tau^2.within, 0.			
**Mean power output**	**Design-specific decomposition of within-designs Q statistic**			
	Placebo: Moderate-dose capsules	14.22	19	0.7710
	Placebo: Low-dose capsules	2.89	7	0.8947
	Placebo: Moderate-dose gum	0.05	2	0.9733
	Placebo: Low-dose gum	0.19	4	0.9957
	Q statistic to assess consistency under the assumption of a full design-by-treatment interaction random effects model: between designs Q statistic, 0.00; degree of freedom, 0; p-value, 0.00; tau. within, 0; tau^2.within, 0.			

Supplement S7: Intransitivity assessments

	$\dot{V}O_{2max}$	Age	Caffeine withdrawal	Sample size
Low-dose capsules	56.18 ± 4.84	25.36 ± 4.17	43.15 ± 19.77	12.54 ± 1.96
Moderate-dose capsules	55.22 ± 5.01	30.63 ± 6.56	34.06 ± 10.41	13.03 ± 5.82
Low-dose gum	62.10 ± 8.71	28.00 ± 6.08	24.00 ± 0.00	12.66 ± 2.08
Moderate-dose gum	49.63 ± 9.27	26.25 ± 5.87	31.15 ± 9.50	13.81 ± 7.96
Low-dose mouth rinse	54.49 ± 0.00	21.00 ± 1.41	24.00 ± 0.00	13.50 ± 2.12
Moderate-dose mouth rinse	50.76 ± 11.37	27.67 ± 5.85	32.00 ± 13.85	8.33 ± 1.52
Placebo	54.49 ± 6.78	28.34 ± 6.30	34.25 ± 12.85	12.85 ± 5.45
P-value	$P = 0.078$	$P = 0.093$	$P = 0.156$	$P = 0.872$

Data is presented as mean ± standard deviation.

Supplement S8: Inconsistency (incoherence) assessments

Completion time								
Comparison	No.Studies	NMA	Direct	Indirect	Difference	Diff 95CI lower	Diff 95CI upper	P-value
Low-dose capsules: Low-dose gum	0	0	NA	0	NA	NA	NA	NA
Low-dose capsules: Low-dose mouth rinse	0	-0.08	NA	-0.08	NA	NA	NA	NA
Low-dose capsules: Moderate-dose capsules	0	-0.03	NA	-0.03	NA	NA	NA	NA
Low-dose capsules: Moderate-dose gum	0	-0.05	NA	-0.05	NA	NA	NA	NA
Low-dose capsules: Moderate-dose mouth rinse	0	-0.34	NA	-0.34	NA	NA	NA	NA
Low-dose capsules: Placebo	9	-0.34	-0.34	NA	NA	NA	NA	NA
Low-dose gum: Low-dose mouth rinse	0	-0.08	NA	-0.08	NA	NA	NA	NA
Low-dose gum: Moderate-dose capsules	0	-0.03	NA	-0.03	NA	NA	NA	NA
Low-dose gum: Moderate-dose gum	0	-0.05	NA	-0.05	NA	NA	NA	NA
Low-dose gum: Moderate-dose mouth rinse	0	-0.34	NA	-0.34	NA	NA	NA	NA
Low-dose gum: Placebo	4	-0.34	-0.34	NA	NA	NA	NA	NA
Low-dose mouth rinse: Moderate-dose capsules	0	0.05	NA	0.05	NA	NA	NA	NA
Low-dose mouth rinse: Moderate-dose gum	0	0.03	NA	0.03	NA	NA	NA	NA
Low-dose mouth rinse: Moderate-dose mouth rinse	0	-0.26	NA	-0.26	NA	NA	NA	NA

Low-dose mouth rinse: Placebo	1	-0.26	-0.26	NA	NA	NA	NA	NA
Moderate-dose capsules: Moderate-dose gum	2	-0.01	0	-0.02	0.02	-0.61	0.64	0.96
Moderate-dose capsules: Moderate-dose mouth rinse	0	-0.31	NA	-0.31	NA	NA	NA	NA
Moderate-dose capsules: Placebo	35	-0.31	-0.31	-0.46	0.15	-0.98	1.28	0.8
Moderate-dose gum: Moderate-dose mouth rinse	0	-0.29	NA	-0.29	NA	NA	NA	NA
Moderate-dose gum: Placebo	8	-0.3	-0.28	-0.44	0.15	-0.82	1.13	0.76
Moderate-dose mouth rinse: Placebo	3	0	0	NA	NA	NA	NA	NA
**Mean power output**								
Comparison	No.Studies	NMA	Direct	Indirect	Difference	Diff 95CI lower	Diff 95CI upper	P-value
Low-dose capsules: Low-dose gum	0	0.16	NA	0.16	NA	NA	NA	NA
Low-dose capsules: Low-dose mouth rinse	0	-0.01	NA	-0.01	NA	NA	NA	NA
Low-dose capsules: Moderate-dose capsules	0	0.08	NA	0.08	NA	NA	NA	NA
Low-dose capsules: Moderate-dose gum	0	0.14	NA	0.14	NA	NA	NA	NA
Low-dose capsules: Moderate-dose mouth rinse	0	0.43	NA	0.43	NA	NA	NA	NA
Low-dose capsules: Placebo	8	0.38	0.38	NA	NA	NA	NA	NA
Low-dose gum: Low-dose mouth rinse	0	-0.16	NA	-0.16	NA	NA	NA	NA
Low-dose gum: Moderate-dose capsules	0	-0.08	NA	-0.08	NA	NA	NA	NA

Low-dose gum: Moderate-dose gum	0	-0.02	NA	-0.02	NA	NA	NA	NA
Low-dose gum: Moderate-dose mouth rinse	0	0.28	NA	0.28	NA	NA	NA	NA
Low-dose gum: Placebo	5	0.22	0.22	NA	NA	NA	NA	NA
Low-dose mouth rinse: Moderate-dose capsules	0	0.09	NA	0.09	NA	NA	NA	NA
Low-dose mouth rinse: Moderate-dose gum	0	0.15	NA	0.15	NA	NA	NA	NA
Low-dose mouth rinse: Moderate-dose mouth rinse	0	0.44	NA	0.44	NA	NA	NA	NA
Low-dose mouth rinse: Placebo	1	0.39	0.39	NA	NA	NA	NA	NA
Moderate-dose capsules: Moderate-dose gum	0	0.06	NA	0.06	NA	NA	NA	NA
Moderate-dose capsules: Moderate-dose mouth rinse	0	0.35	NA	0.35	NA	NA	NA	NA
Moderate-dose capsules: Placebo	20	0.3	0.3	NA	NA	NA	NA	NA
Moderate-dose gum: Moderate-dose mouth rinse	0	0.29	NA	0.29	NA	NA	NA	NA
Moderate-dose gum: Placebo	3	0.24	0.24	NA	NA	NA	NA	NA
Moderate-dose mouth rinse: Placebo	1	-0.05	-0.05	NA	NA	NA	NA	NA

Supplement S9: Forest plot of all studies

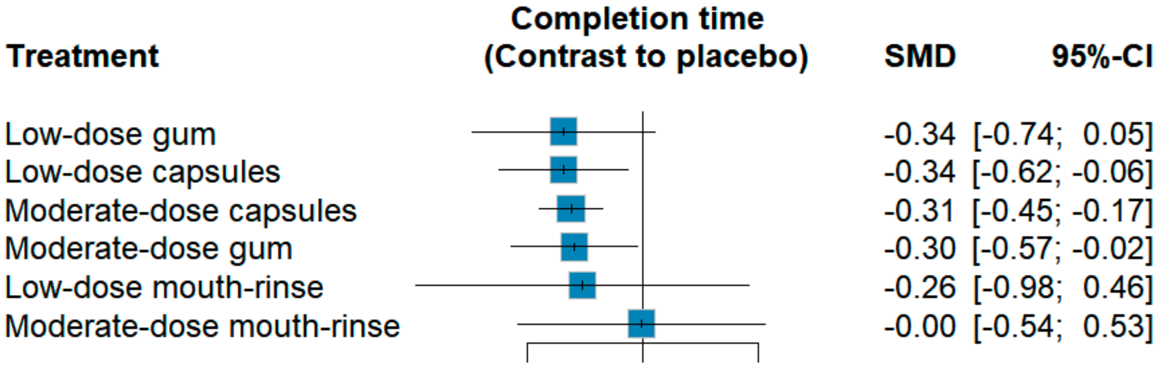

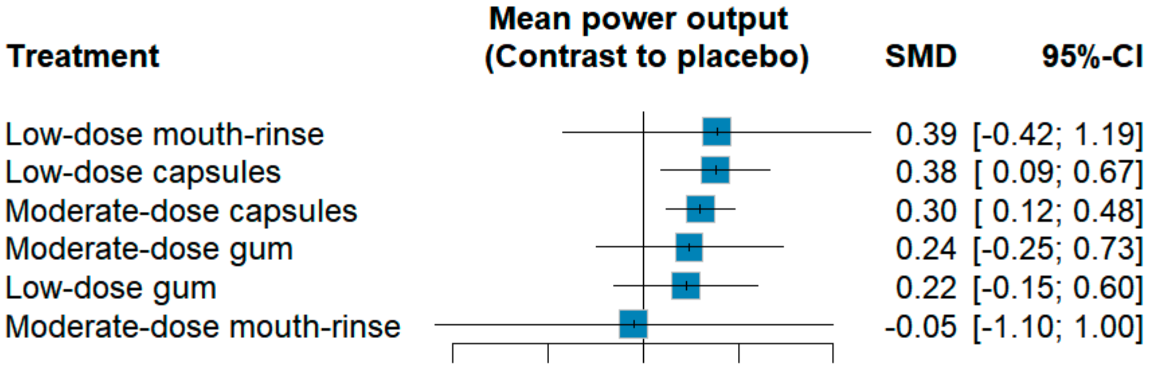

Supplement S10: Comparison-adjusted funnel plots

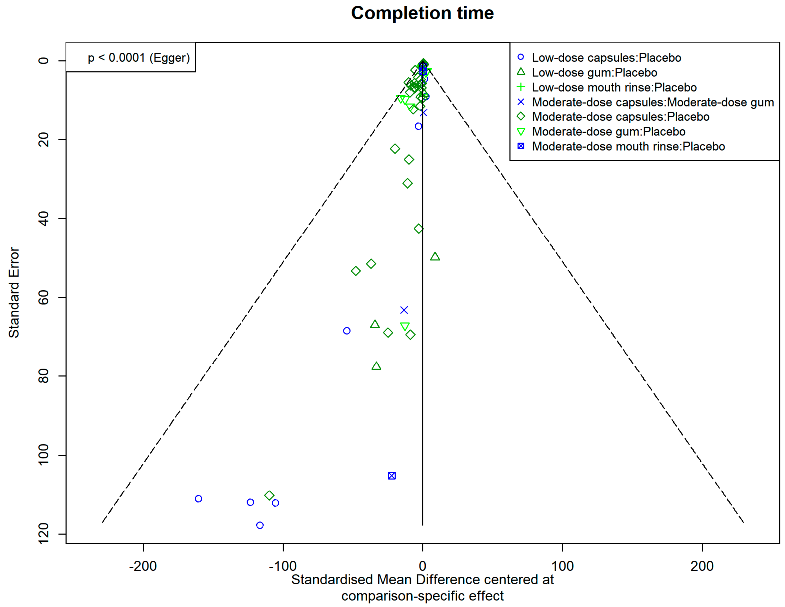

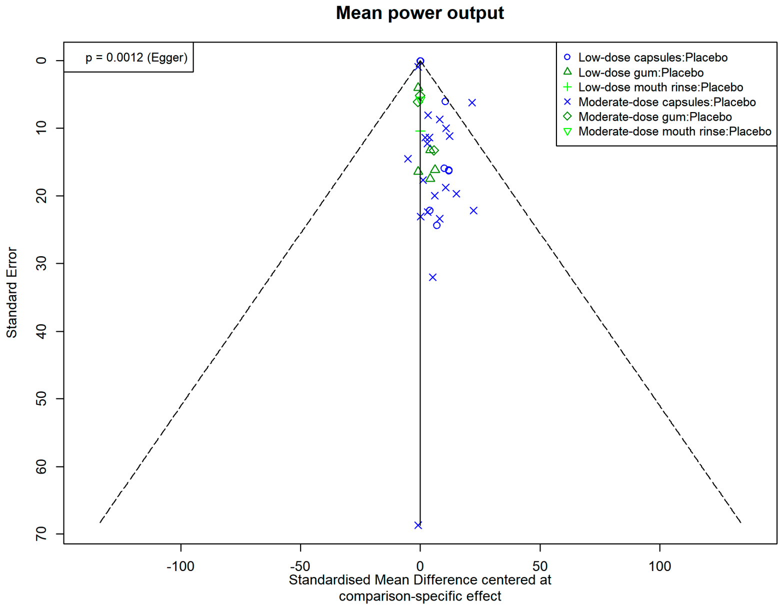

Supplement S11: Trim and fill analysis

Outcome indicators	Potential number of missing studies	Variables	Pooled estimate	95% CI	
				Lower Limit	Upper Limit
Time	4	Observed values	-0.287	-0.393	-0.182
		Adjusted values	-0.309	-0.412	-0.206
MPO	0	Observed values	0.287	0.152	0.421
		Adjusted values	0.287	0.152	0.421

CI: confidence intervals, MPO: mean power output, Time: completion time.

Supplement S12: Network meta-regression

Network meta-regression analyses of completion time and mean power output outcome were performed to investigate potential moderating variables. The network meta-regression model was fitted in a Bayesian framework using the Markov chain Monte Carlo (MCMC) method in the R statistical package "Gemtc". Four chains were run using a non-informative prior. The number of iterations for each chain was 25,000, with the first 5,000 iterations discarded.

Covariate	Completion time				
	DIC	pD	Residual Deviance	Shared beta (Median and 95% CrI)	SD
Age	32.9	8.4	24.5	0.04 (-0.21, 0.29)	0.12
$\dot{V}O_{2max}$	32.7	8.3	24.4	-0.02 (-0.29, 0.25)	0.14
Exercise distance	31.8	7.7	24.0	0.09 (-0.14, 0.34)	0.12
Exercise duration	32.1	7.9	24.2	0.06 (-0.18, 0.30)	0.12
Ingestion timing	28.7	5.6	23.1	0.19 (-0.13, 0.52)	0.12
Habitual caffeine intake	33.1	8.5	24.6	-0.01 (-0.26, 0.25)	0.13
Exercise mode	32.7	8.3	24.4	0.03 (-0.19, 0.27)	0.12
**Covariate**	**mean power output**				
	**DIC**	**pD**	**Residual Deviance**	**Shared beta (Median and 95% CrI)**	**SD**
Age	32.0	8.6	23.4	-0.09 (-0.44, 0.25)	0.17
$\dot{V}O_{2max}$	32.6	9.1	23.5	-0.01 (-0.47, 0.44)	0.23
Exercise distance	30.8	7.8	23.0	-0.15 (-0.57, 0.26)	0.21
Exercise duration	30.6	7.7	22.9	-0.16 (-0.55, 0.22)	0.19
Ingestion timing	28.7	6.0	22.6	-0.26 (-0.82, 0.29)	0.28
Habitual caffeine intake	28.0	6.4	21.5	-0.22 (-0.54, 0.09)	0.16
Exercise mode	32.5	8.9	23.6	0.03 (-0.31, 0.37)	0.17

Note: CrI, credible interval. DIC, deviance information criterion. SD, standard deviation. $\dot{V}O_{2max}$: maximal oxygen uptake
